# Believing and Beliefs—Neurophysiological Underpinnings

**DOI:** 10.3389/fnbeh.2022.880504

**Published:** 2022-04-19

**Authors:** Rüdiger J. Seitz

**Affiliations:** Department of Neurology, Centre of Neurology and Neuropsychiatry, LVR-Klinikum Düsseldorf, Medical Faculty, Heinrich-Heine-University Düsseldorf, Düsseldorf, Germany

**Keywords:** belief, fMRI, perception, valuation, language, representations, propositions

## Introduction

The credition model posits that beliefs are the result of neural processes that involve the perception of external information and their valuation in terms of personal meaning determining a person's behavioral decisions (Seitz et al., 2018). These processes of believing typically evolve in a pre-linguistic fashion and include memory functions by which beliefs can be stored and recalled (Seitz et al., 2022). Thus, beliefs are fundamental representations of imaginative and emotional content that link an individual's prior experience with his/her future behavior. Importantly, people can become aware of what they believe and express it explicitly by “I believe …” (Oakley and Halligan, 2017; Seitz and Angel, 2020). Such propositions have a first-person perspective and can communicate the subject's certainty or trust into such a personally held belief to other people.

In this communication, the brain structures related to the processes of believing as identified by functional imaging are described. In the first part, imaging studies are presented in which healthy subjects processed statements of believing. The second part focuses on functional MRI studies addressing pre-linguistic processing involved in belief formation and updating.

## Verbal Processing Underlying Believing

**Figure 1:**
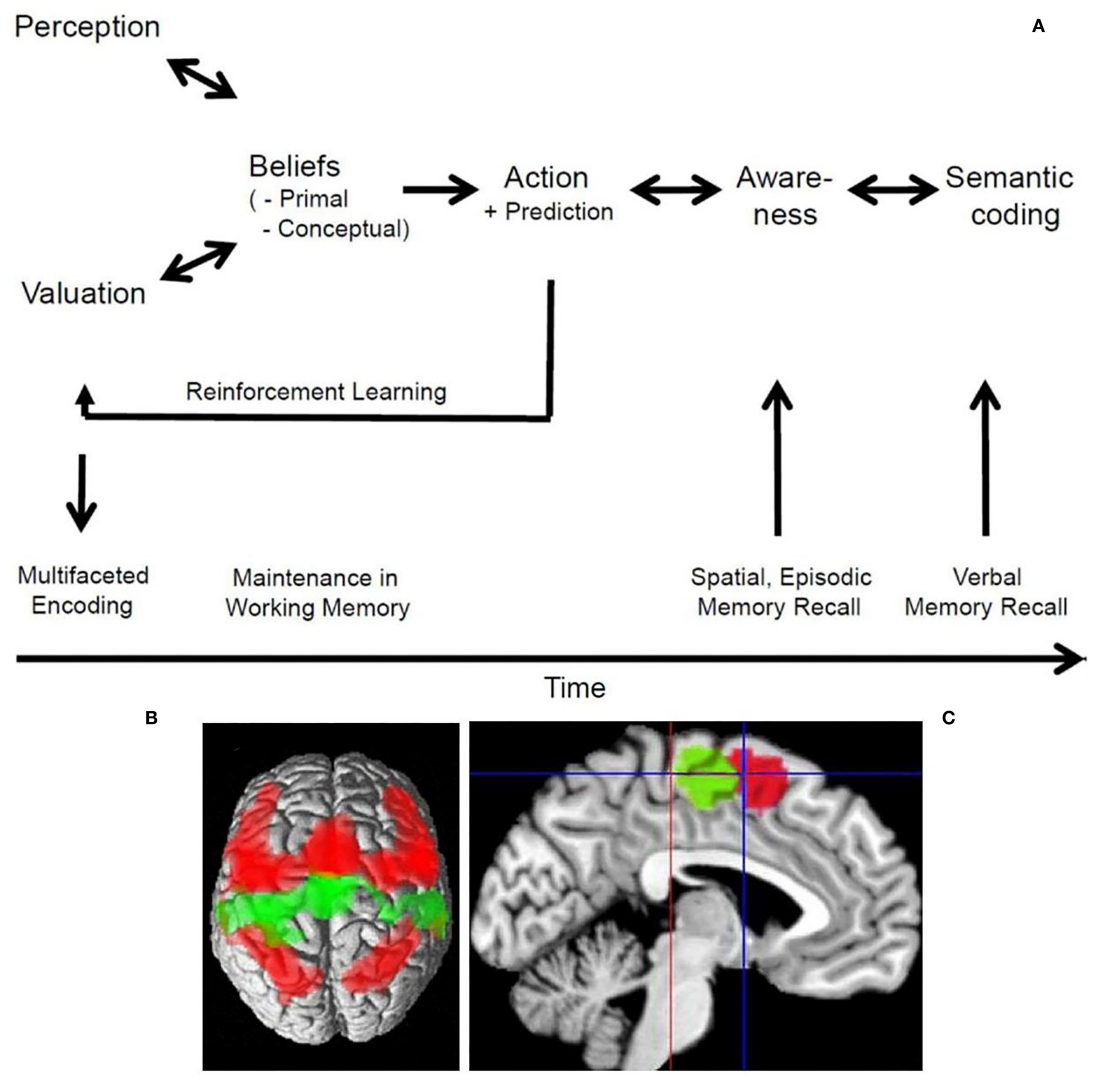
The processes of believing and the dorsal medial frontal cortex. **(A)** Schematic drawing of the processes of believing and their close relation to memory functions. The bi-directional arrows account for putative bottom-up and top-down processing, while the uni-directional arrows indicate the major flow of information. Further details in Seitz et al. (2022). **(B)** The connectivity patterns of the SMA (green) and pre-SMA (red) as evident from a meta-analysis of functional imaging studies in a dorsal view on the brain. The SMA is predominantly connected bilaterally with the motor cortex, while the pre-SMA is predominantly connected bilaterally with the dorsolateral prefrontal as well as inferior and superior parietal cortex. Note, that the pre-SMA projects also to the SMA. **(C)** Medial aspect of the brain with cytoarchitectonic localization of the SMA (green) posterior and of the pre-SMA (red) anterior to the vertical plane through the anterior commissure (blue line); red line indicates the vertical plane through the posterior commissure. Further details in Ruan et al. (2018). To **(B,C)** the Creative Commons Attribution 4.0 International License (http://creativecommons.org/licenses/by/4.0/) applies.

Secular political beliefs and religious beliefs are based on narratives that can be communicated by recital of stories or by written manifests. Ritual acts associated with these narratives lend emotional flavor to them by cognitive-emotional integration. Such beliefs correspond to so-called conceptual beliefs (Figure 1A). The first imaging study addressing the question which brain structures are involved in processing of a religious belief was by Azari et al. (2001). Christian Protestants were subjected to functional imaging while they recited Psalm 23. The strongest activation occurred in dorsal medial frontal cortex in comparison to reciting a nursery as well as to non-believing subjects (Figures 1B,C). Note, that in this study the neural representations of the Christian belief content was the research topic. This is different from the following three studies in which first-person assessments of believing were studied.

In one study, healthy subjects were required to indicate whether they agreed to statements about the involvement of God in the world such as “God protects one's life” (Kapogiannis et al., 2009). The pattern of activation involved also the dorsal medial frontal cortex besides a number of other cortical areas. It was suggested that the subjects engaged mentalizing processes to understand God's intent (Kapogiannis et al., 2009). A subsequent multivariate directional connectivity analysis showed that the religious subjects preferentially activated a pathway from inferolateral to dorsal medial frontal cortex. This pattern was interpreted as monitoring of the intent and involvement of supernatural agents. In contrast, perception of supranatural agents was found to engage pathways involved in fear regulation and affective mentalizing (Kapogiannis et al., 2014).

In a more recent functional magnetic resonance imaging (fMRI) study healthy subjects were asked to decide whether propositions presented to them were true or false. These included statements that can be tested such as “I believe that hamsters are more common as pets than turtles”. And there were statements that cannot be tested such as “I believe that giving love to others is the most important thing in my life”. These assessments involved widespread, but non-overlapping cortical circuits (Howlett and Paulus, 2015). The dorsomedial prefrontal cortex, the precuneus and the cingulate gyrus were activated when the subjects were certain concerning their assessments of the testable statements, while the superior temporal gyrus was activated when the subjects were certain concerning the non-testable statements. More recently, Chinese and Danish students were required to indicate in a yes-no response if they believed that adjectives presented to them described themselves, celebrities or had positive or negative valence. The behavioral data showed cultural group differences in self-construal, self-believing and celebrity-believing judgments. The fMRI data showed that there were common activations as well as significant differences across both groups of participants. Importantly, the dorsal medial frontal cortex was activated in the Chinese but not Danish students with regard to self-construal (Gao et al., 2022).

## Pre-linguistic Processes of Believing

The formation and updating of beliefs involve rapidly evolving neural processes such as perception, valuation, sensorimotor control, mentalizing, and perceptive-emotional integration. These are called primal beliefs or belief precursors and do not depend on language functions (Oakley and Halligan, 2017; Seitz and Angel, 2020). Conversely, people can state their primal or pre-linguistic beliefs verbally only after they have become aware of them. Inherent in these processes is the notion of the subjective first-person perspective of valuating of external information in terms of personal meaning and relevance. These representations have an imaginative character and are continuously updated by new information (Figure 1A). They build the basis on which subjects generate their spontaneous actions and make predictions of future events. These processes are maintained in putative parallel cortico-subcortical loops in the brain which was taken as basis for computational modeling of belief formation (Friston et al., 2017). From a methodological point of view the instructions to perform the tasks in functional imaging experiments were verbal statements. However, the neuropsychic processes initiated by them did not depend on language functions. Thus, the functional imaging studies addressed the question which structures of the human brain are engaged in relation to such pre-linguistic processes of believing. They are summarized here as follows.

The neural coding of emotional valence has been shown to involve widespread neural circuits distributed over different cortical and subcortical regions. The dorsolateral prefrontal cortex has been shown to be deeply interwoven with the integration of emotion and cognition (Gray et al., 2002; Okon-Singer et al., 2015). This also applies to affective and cognitive perspective taking (Healey and Grossman, 2018). Moreover, the prefrontal cortex and posterior cingulate direct attention to processes of unconscious threat (Etkin et al., 2009), while the right lateral prefrontal cortex was found to be involved in preference judgments (Elliott and Dolan, 1998). In addition, the basolateral amygdala and the nucleus accumbens are important brain structures related to the diversified aspects of valence encoding (Le Doux, 1996; Namburi et al., 2016; Vestergaard and Schultz, 2020). Likewise, it was found that a well-coordinated prefrontal-striatal network that is activated while a subject is experiencing a reward shapes preferences for future choices (Tanaka et al., 2020). Also, cognitive appraisal of emotions, belief updating, and self-perspective inhibition has been related to activity in a right fronto-parietal network (Miura et al., 2020). As a consequence, the lateral prefrontal cortex participates in the dynamic control of executive actions and in behavioral control (Mansuri et al., 2009). Importantly, positive and negative outcomes are encoded in the medial prefrontal cortex but with opposite signs in its ventral and dorsal subdivisions (Pischedda et al., 2020).

Besides its role in integrating cognitive and emotional information, the prefrontal cortex has been shown to be involved also in maintaining the concept of a personal self (Fossati et al., 2003). Specifically, activity in the dorsolateral prefrontal cortex was found in a phonological or semantic judgment task to be associated with priming effects (Lau and Passingham, 2007). Moreover, it was found that visually presented personally relevant words that signal important emotional clues engage a widely distributed set of brain regions including the dorsal medial and lateral prefrontal cortex (Huth et al., 2016). Further, emotion-denoting words were found to activate a large-scale neural network in the prefrontal cortex subserving the affective dimensions of valence and another network involving the left parahippocampus and dorsal anterior cingulate for affective arousal (Posner et al., 2009). Importantly, these processes did not activate language-related cortical areas.

Processing of events in the environment involves the dorsal cerebral midline structures including the supplementary motor area (SMA) and pre-SMA (Figure 1C). For example, when the cingulate is activated, it is likely that a negative event occurred (Jocham et al., 2009). This may be related to the time needed and effort invested to resolve a conflict (Kennerley et al., 2008; Mansuri et al., 2009). Also, during the generation and control of behavior, subliminal stimuli are thought to trigger inhibitory processes in extended prefrontal cortical areas that act on the pre-supplementary motor area (van Gaal et al., 2008). Notably, it has been found that anticipation of reward and punishment are mediated by opponent mechanisms but have some shared activations (Lake et al., 2019). Furthermore, activation of the orbitofrontal cortex reflects the subjective value of anticipated outcomes, whereas activation of the SMA reflects the probability of a persons' choice (FitzGerald et al., 2009). In contrast, activity in a cortico-subcortical network involving the striatum and the pre-SMA was found to be related to reward prediction (Hsu et al., 2009). Interestingly, involvement of the pre-SMA and bilaterally of the insula reflected subjective uncertainty (FitzGerald et al., 2009).

As humans develop subjective preferences and are able to make predictions about future events and other people's behavior, they need to decide what to do next, how to react to the actions of other people, and how to maximize the benefit between differential choices. Typically, these decisions can lead to either an immediate reward or to long-term satisfaction (Rolls, 2006). An interesting question is whether such choices require conscious awareness. Perceptual decisions have been found to be based on the matching of predicted and observed evidence in tests of perceptually ambiguous stimuli (Summerfield et al., 2006). Subjective preference judgments are mediated by the prefrontal cortex, medial orbitofrontal cortex, insula, and cingulate (Chaudhury et al., 2009). It was shown experimentally that people make choices via the anterior prefrontal cortex using preferences of which they are not aware (Tusche et al., 2010). Similarly, day-to-day decisions were found to involve the ventromedial prefrontal related to valuation and choice (Levy and Glimcher, 2012; Kumar et al., 2019; Koscik et al., 2020). Decisions concerning reasoning about other peoples face expressions were shown to be made with high accuracy in a time window too little to account for conscious awareness (Prochnow et al., 2013). Nevertheless, the entire cortical processing network related to emotional face perception was involved. In contrast, the fusiform face area was more active during supraliminal face presentation. This corresponded to the observations that brain regions, including the amygdala, become activated by emotional faces only when sufficient attentional resources concerning the effects of valence are available (Pessoa et al., 2002). Interestingly, observing people interacting with each other activated the posterior superior temporal cortex related to meta-theoretical inference about what is being observed (Isik et al., 2017). It is of note that the pre-SMA was involved in such decisions (Prochnow et al., 2014). Therefore, it can be argued that the pre-SMA integrates online information processing in the dorsolateral prefrontal cortex with motor command processing (Figure 1B). This is consistent with the observation that preference adjustments in difficult decisions are related to activity in a widespread left dorsolateral prefrontal-midparietal network (Voigt et al., 2019). Such, findings support the view that computation of the expected value in mesolimbic structures represents an affective component, whereas cortical regions represent a probabilistic component, and may integrate the two (Knutson et al., 2005).

## Discussion

Beliefs are pre-linguistic representations of imaginative and emotional content that link an individual's prior experience with his/her future behavior. These functions enable humans to infer social meaning from other people's behavior and to make corresponding attributions (Malle and Korman, 2022). Furthermore, humans can become aware of their beliefs and express their content in the form of semantic expressions. It was shown here that processing of beliefs engages widespread cortical circuits related to inferential attribution, cognitive-emotional integration, and language functions. The dorsal medial frontal cortex comprising the so-called pre-SMA was shown to be a critical hub with a large-scale cortico-subcortical loop involving the thalamus and reciprocal connectivity to prefrontal and parietal cortical areas (Reid et al., 2015; Ruan et al., 2018). The overlap of this connectivity pattern with the cortical circuitry related to working memory and the so-called default network (Reid et al., 2015) accords with a prominent role also in belief evaluation (Sugiura et al., 2015). Belief evaluation is a language-based function by which humans can consider critically what they believe and how this corresponds to their predictions (Coltheart et al., 2011). Conversely, patients with neurological and psychiatric diseases provided evidence that focal brain lesions can interfere with the formation, updating and evaluation of beliefs (Coltheart et al., 2011; Seitz, 2022). Thus, brain diseases interfering with the processes of believing can induce abnormal beliefs that can cause deviant behavior.

## Author Contributions

The author confirms being the sole contributor of this work and has approved it for publication.

## Funding

This study was funded by the Volkswagen Foundation: #99835, Siemens Healthineers, and the Betz Foundation. Siemens Healthineers were not involved in the study design, collection, analysis, interpretation of data, the writing of this article or the decision to submit it for publication.

## Conflict of Interest

The author declares that the research was conducted in the absence of any commercial or financial relationships that could be construed as a potential conflict of interest.

## Publisher's Note

All claims expressed in this article are solely those of the authors and do not necessarily represent those of their affiliated organizations, or those of the publisher, the editors and the reviewers. Any product that may be evaluated in this article, or claim that may be made by its manufacturer, is not guaranteed or endorsed by the publisher.
